# Impacts of Ultrasonic Treatment for Black Soybean Okara Culture Medium Containing Choline Chloride on the β-Glucosidase Activity of *Lactiplantibacillus plantarum* BCRC 10357

**DOI:** 10.3390/foods12203781

**Published:** 2023-10-14

**Authors:** Chia-Min Wu, Chun-Yao Yang

**Affiliations:** Department of Food Science, Fu Jen Catholic University, No. 510, Zhongzheng Rd., Xinzhuang District, New Taipei City 242062, Taiwan; tiannait0901@gmail.com

**Keywords:** ultrasound, choline chloride, lactic acid bacteria, fermentation, β-glucosidase, black soybean okara

## Abstract

The effects of ultrasonic treatment for the culture medium of solid black soybean okara with choline chloride (ChCl) on the survival and β-glucosidase activity of *Lactiplantibacillus plantarum* BCRC 10357 (*Lp*-BCRC10357) were investigated. A mixture of 3% dried black soybean okara in de Man–Rogosa–Sharpe (*w*/*v*) was used as the Oka medium. With ultrasonic treatment (40 kHz/300 W) of the Oka medium at 60 °C for 3 h before inoculation, the β-glucosidase activity of *Lp*-BCRC10357 at 12 h and 24 h of incubation amounted to 13.35 and 15.50 U/mL, respectively, which was significantly larger than that (12.58 U/mL at 12 h and 2.86 U/mL at 24 h) without ultrasonic treatment of the medium. This indicated that ultrasonic treatment could cause the microstructure of the solid black soybean okara to be broken, facilitating the transport of ingredients and *Lp*-BCRC10357 into the internal structure of the okara for utilization. For the effect of ChCl (1, 3, or 5%) added to the Oka medium (*w*/*v*) with ultrasonic treatment before inoculation, using 1% ChCl in the Oka medium could stimulate the best response of *Lp*-BCRC10357 with the highest β-glucosidase activity of 19.47 U/mL in 12 h of incubation, showing that *Lp*-BCRC10357 had a positive response when confronting the extra ChCl that acted as an osmoprotectant and nano-crowder in the extracellular environment. Furthermore, the Oka medium containing 1% ChCl with ultrasonic treatment led to higher β-glucosidase activity of *Lp*-BCRC10357 than that without ultrasonic treatment, demonstrating that the ultrasonic treatment could enhance the contact of ChCl and *Lp*-BCRC10357 to regulate the physiological behavior for the release of enzymes. In addition, the analysis of the isoflavone content and antioxidant activity of the fermented product revealed that the addition of 1% ChCl in the Oka medium with ultrasonic treatment before inoculation allowed a higher enhancement ratio for the biotransformation of isoflavone glycosides to their aglycones, with a slight enhancement in the antioxidant activity at 24 h of fermentation. This study developed a methodology by combining ultrasonic treatment with a limited amount of ChCl to allow the culture medium to acclimate *Lp*-BCRC10357 and release high levels of β-glucosidase, and this approach has the potential to be used in the fermentation of okara-related products as nutritional supplements in foods.

## 1. Introduction

Soybean okara (residue), as an insoluble by-product in the production of soy-derived products in the food industry, is rich in dietary fiber and nutritional compounds beneficial to human health, and it is worth reusing in foods for valorization or as a nutritional supplement in feed [1,2,3,4]. Black soybean okara is the residue derived from producing black soymilk from black soybean (*Glycine max* (L.) Merr.), which contains functional compounds such as carotenoids, saponins, and isoflavones, with benefits such as anti-aging, anti-inflammatory, and detoxification activity and relieving kidney disease [5,6]. During the processing of soymilk, about 12–30% of the isoflavones in soybeans can be retained in the okara [7], within which the content of bioactive isoflavone aglycones can be increased via the biotransformation of their glycosides for use in foods [8]. Such biotransformation can be achieved using the enzyme β-glucosidase, released from lactic acid bacteria (LAB), which has benefits for the host, such as preventing cancer and inhibiting intestinal pathogens, and it can be used in the fermentation of okara [9,10,11]. Wang et al. (2022) reported that the use of enzymatic hydrolysis and fermentation had a positive influence on the nutritional and functional profile of the okara, and a greater extent of hydrolysis facilitated the growth of *Lactiplantibacillus plantarum* during the fermentation of okara [12].

However, when using LAB to ferment the solid okara, the much easier transport of the ingredients and enzyme, as well as the bacteria, into the internal structure of the solid okara would be more advantageous in the extraction and biotransformation of the isoflavones and other functional ingredients. Modifying the surface structure of the okara helps to increase the portions exposed to the bacteria for utilization. Lin et al. (2020) investigated the effects of fermentation and microwave treatment on the structure and functional properties of okara dietary fiber, showing that the honeycomb structure of the okara dietary fiber was more obvious and the crystal structures were slightly damaged after fermentation and microwave treatment [13]. Besides microwave treatment, ultrasound can be an effective method to modify the structure of the okara. Ultrasound, as a non-thermal green technology, can induce a cavitation effect in the liquid medium to generate microstreaming and local hotspots to enhance the chemical reaction and facilitate the mass transfer of components [14]. Using ultrasound at different temperatures to extract the residual proteins from the okara by-product was reported to modify the secondary and tertiary protein structures in a significant way [15]. Ultrasonic treatment of solid okara could be beneficial to enhance the openness of the microstructure for fermentation with LAB [16]. 

Among the LAB groups, *L. plantarum* shows physiological behavior with a highly adaptive response when encountering various kinds of environmental stress, such as the stress from ultrasound and different nutrients [17,18], and is widely applied in the food industry [19,20,21]. During fermentation or in food production, LAB may experience abiotic and biotic stresses, including osmotic stress [22]. The increase in osmolarity presents a challenge in the production of fermented foods, and the positive turgor of bacterial cells is decreased due to dehydration from the effect of osmotic stress [22]. Some molecules that can act as osmoprotectants (e.g., glycine betaine, choline, and proline) could be employed to balance the difference between intracellular and extracellular osmolarity for rehydration [22,23,24]. 

Choline is essential in the synthesis of phospholipids in cell membranes, and it is converted to betaine and used as an osmolyte as well as a methyl donor [25]. Kets et al. (1997) investigated the physiological response of *L. plantarum* subjected to osmotic stress from NaCl by adding betaine, choline, or acetylcholine in the growth medium; they found that the three compounds were able to counterbalance the negative effects of NaCl on the growth rate, and it was not vital for the anionic compounds sulfate, chloride, and phosphate in balancing the intracellular charge in *L. plantarum* [26]. Choline chloride ((2-hydroxyethyl) trimethylammonium chloride), a compound with a choline cation and chloride anion, is often used as a nutritional supplement in animal feeds [27]. The aqueous choline chloride solution has been reported to be an alternative to deep eutectic solvents in some industrial applications, and choline chloride can serve as a protecting co-solvent for proteins to restrict urea in approaching the protein surface and maintain the water structure around the protein [28]. For the fermentation of solid okara with *L. plantarum*, the presence of choline chloride in the culture medium under different intensities of ultrasonic irradiation could be utilized to influence the survival of *L. plantarum* because of the change in the environment, such that *L. plantarum* might be induced to regulate its physiological behavior to adapt to the new environment, thus releasing different levels of β-glucosidase enzyme.

Therefore, various environmental niches could be suitably applied to acclimate *L. plantarum* with a positive stress response, so as to be used in the fermentation of foods. In this study, the aim was to investigate the effects of ultrasonic treatment for the black soybean okara culture medium with the addition of choline chloride on the β-glucosidase activity of *L. plantarum* BCRC 10357. Various conditions of ultrasonic treatment of the medium of de Man–Rogosa–Sharpe (MRS) with dried black soybean okara were performed to find the most favorable conditions for the growth of *L. plantarum* BCRC 10357. The influences of different amounts of choline chloride in the medium with ultrasonic treatment on the stress response of *L. plantarum* BCRC 10357 and various modes of ultrasonic treatment on the culture system were also assessed. The antioxidant activity and the biotransformation of isoflavone glycosides into their aglycones in the fermented product of black soybean okara were analyzed. In addition, the surface morphologies of the solid parts separated from the fermented medium for different conditions of ultrasonic treatment and choline chloride were analyzed.

## 2. Materials and Methods

### 2.1. Materials

The chemical reagents, *p*-nitrophenyl β-D-glucopyranoside (*p*-NPG), *p*-nitrophenol (*p*-NP), Folin–Ciocalteu phenol reagent (2N), (±)-6-hydroxy-2,5,7,8-tetramethylchromane-2-carboxylic acid (Trolox), choline chloride (ChCl), ferric chloride, 2,4,6-tris(2-pyridyl)-s-triazine (TPTZ), daidzein, daidzin, genistein, and genistin were purchased from Sigma-Aldrich, Merck KGaA (Darmstadt, Germany). The de Man–Rogosa–Sharpe (MRS) was purchased from Becton, Dickinson, and Company (Franklin Lakes, NJ, USA). Other reagents were purchased from Alfa Aesar, Thermo Fisher Scientific (Waltham, MA, USA), Taiwan Sugar Corporation (Tainan City, Taiwan), Bionovas Biotechnology Co., Ltd. (Toronto, ON, Canada), Biomate (Taipei, Taiwan), Sigma-Aldrich, Merck KGaA (Darmstadt, Germany), and Merck (Darmstadt, Germany).

The black soybean (*G. max* (L.) Merr.) (Tainan No. 3, place of origin being Tainan, Taiwan), which was harvested at the end of 2019 and subjected to vacuum packaging for storage, was purchased from Shia Ying Farmers’ Association (Tainan, Taiwan) and used to prepare black soymilk under the conditions of soaking and homogenization, as described in Tseng and Yang (2022) [6]. The residue (black soybean okara) was separated from the black soymilk using filtration and was then freeze-dried and screened with a 60-mesh sieve to obtain the dried black soybean okara for the experiments. 

### 2.2. Culture of L. plantarum BCRC 10357

*L. plantarum* BCRC 10357 (abbreviated as *Lp*-BCRC10357) (other collection no.: ATCC 8014), obtained from the Food Industry Research and Development Institute (Hsinchu, Taiwan), was selected to evaluate the physiological responses under the stresses from ultrasonic treatment and in the presence of ChCl in the medium of MRS broth with solid black soybean okara. The *Lp*-BCRC10357 culture was preserved at −80 °C and activated by inoculation in sterilized MRS broth at the ratio of 1% (*v*/*v*) at 37 °C for 24 h two times. The viable cell counts of *Lp*-BCRC10357 were determined with the pour plate method and 10-fold serial dilution method using sterilized 0.1% (*w*/*v*) peptone water to dilute the fermented liquid, and they were expressed as log CFU/mL.

### 2.3. Ultrasonic Treatment and Fermentation of Black Soybean Okara with Lp-BCRC10357

The culture medium of 3% dried black soybean okara in MRS broth (*w*/*v*) was used as the Oka medium. The Oka medium without ultrasonic treatment was prepared (denoted as Oka (no US)) to evaluate the effect of ultrasonic treatment on the viable cell counts and β-glucosidase activity of *Lp*-BCRC10357. An ultrasonic bath of 40 kHz/300 W (LEO-3002S, Leo Ultrasonic Co., New Taipei City, Taiwan) with a power density of 0.028 W/mL was employed to pretreat the medium. The ultrasonic treatment (40 kHz/300 W) of the Oka medium was carried out at 60 °C for 3 h (denoted as Oka-US) or at 30 °C for 20 min (denoted as Oka-US-L) before inoculation to find the most suitable conditions for the growth of *Lp*-BCRC10357.

To explore the influence of the amount of ChCl in the Oka medium on the survival of *Lp*-BCRC10357, the ultrasonic treatment (40 kHz/300 W) of the medium with x% ChCl in the Oka (*w*/*v*) was performed at 60 °C for 3 h before inoculation and denoted as x%ChCl-US (x = 1, 3, or 5). The medium with 1% ChCl in the Oka without ultrasonic treatment before inoculation was denoted as 1%ChCl (no US) for comparison.

The test medium was sterilized at 121 °C for 20 min. Then, *Lp*-BCRC10357 was inoculated at 1% (*v*/*v*) (about 7 log CFU/mL) into the sterilized medium, and the fermentation was started at 37 °C for 0–48 h. The viable cell counts and β-glucosidase activity were determined. Moreover, fermentation using 1%ChCl-US medium inoculated with the pretreated *Lp*-BCRC10357, which had been treated with an ultrasound probe (SONOPULS HD 4200, BANDELIN Electronic GmbH & Co. KG, Germany) set at 60% amplitude (20 kHz/200 W) and 25 °C for 2 min, was also performed and denoted as 1%ChCl-USP.

At the selected fermentation time, the fermented broth was separated as the supernatant from the medium by centrifugation and then freeze-dried to obtain the dried fermented product (denoted as FP). The FP was further extracted with 80% aqueous methanol at 30 °C for 4 h under 40 kHz/300 W of ultrasound. Then, the mixture was centrifuged at 4 °C using 4000 rpm for 10 min, and the supernatant was freeze-dried to obtain the FP extract (denoted as FPE). 

### 2.4. Determination of β-Glucosidase Activity

According to the method of Tseng and Yang (2022), the determination of the β-glucosidase activity of *Lp*-BCRC10357 was based on the measurement of the quantity of *p*-NP released from the hydrolysis rate of *p*-NPG [6], and it is briefly described in the following. The *p*-NPG was added into the fermented liquid to react at 37 °C for 30 min, and the reaction was stopped by adding Na_2_CO_3_ into the mixture. After centrifugation, the supernatant was separated from the mixture and then filtered and analyzed for β-glucosidase activity using a spectrophotometer at 405 nm (Hitachi, Ratio Beam Spectrophotometer U-5100, Tokyo, Japan).

### 2.5. Determination of Ferric-Reducing Antioxidant Power

The determination of the ferric-reducing antioxidant power (FRAP) of the FPE followed the method of Tsui and Yang (2021) using Trolox equivalent (TRE) [10], and it is briefly described in the following [10]. The liquid sample of FPE was first prepared by dissolving it in 80% aqueous methanol at a ratio of 1:10 (*w*/*v*). Then, the FRAP reagent was mixed with the liquid sample of FPE and deionized water to react at 37 °C for 4 min. The FRAP was determined by measuring the absorbance of the mixture using a spectrophotometer at 593 nm, and it was expressed as μg-TRE/g-FPE or further μg-TRE/g-FP.

### 2.6. HPLC Analysis of Isoflavones

High-performance liquid chromatography (HPLC) was employed to determine the isoflavone content in the FPE using daidzin, daidzein, genistin, and genistein (Sigma-Aldrich) as the standards for quantification. The liquid sample of FPE for analysis was prepared as stated in Section 2.5. The HPLC system was equipped with a UV–VIS detector (Hitachi Chromaster 5420 UV–VIS detector, Hitachi, Ltd., Tokyo, Japan) at 260 nm and a Mightysil RP-18 column (5 μm, 250 mm × 4.6 mm, Kanto Chemical Co., Tokyo, Japan). The HPLC conditions followed the method of Yu and Yang (2019) with a slight modification [16]. The mobile phase of 0.1% (*v*/*v*) trifluoroacetic acid (solvent A) and acetonitrile (solvent B) (Merck, Darmstadt, Germany) was set at a flow rate of 0.8 mL/min with the gradient of solvent A as follows: 90% at 0–10 min, 90–45% at 10–35 min, 45–90% at 35–45 min, and 90% at 45–55 min.

### 2.7. FE-SEM Analysis

The surface morphology of the solid parts that were separated from the fermented medium after fermentation was analyzed using a field emission scanning electron microscope (FE-SEM) (JEOL, JSM-7800F, Tokyo, Japan). 

### 2.8. Statistical Analysis

Each experiment was performed three times using three independent samples for each condition, and the experimental data were expressed as the mean ± standard deviation (*n* = 3). The statistical analysis was performed by applying one-way ANOVA with Duncan’s multiple range test using IBM SPSS Statistics 20 (IBM SPSS Statistics for Windows v. 20.0, IBM Corp, Armonk, NY, USA). A significant difference was determined at *p* < 0.05.

## 3. Results and Discussion

### 3.1. Ultrasonic Treatment of the Medium of MRS with Black Soybean Okara

**Figure 1:**
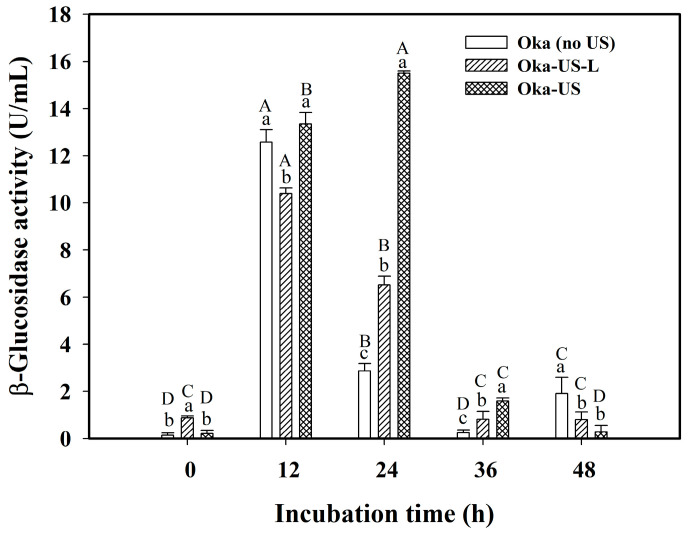
Variations in β-glucosidase activity of *L. plantarum* BCRC 10357 incubated at 37 °C for 48 h using different conditions of ultrasonic treatment (40 kHz/300 W) for the medium of MRS with 3% black soybean okara (Oka). Oka (no US): the Oka medium without ultrasonic treatment; Oka-US-L: the Oka medium with ultrasonic treatment at 30 °C for 20 min before inoculation; Oka-US: the Oka medium with ultrasonic treatment at 60 °C for 3 h before inoculation. Data were expressed as mean ± standard deviation with triplicate experiments (*n* = 3). Different superscript lowercase letters at the same incubation time and different superscript uppercase letters at the same conditions with or without ultrasonic treatment were significantly different (*p* < 0.05) by Duncan’s multiple range test.

The viable cell counts and β-glucosidase activity of *Lp*-BCRC10357 incubated at 37 °C for 48 h under different conditions of ultrasonic treatment (40 kHz/300 W) of the Oka medium are shown in Table 1 and Figure 1, respectively. The viable cell counts of *Lp*-BCRC10357 were greater than 9.4 log CFU/mL in 12–24 h of incubation under different conditions of ultrasonic treatment for the Oka medium (Table 1). Among them, the Oka medium with ultrasonic treatment at 60 °C for 3 h before inoculation (Oka-US) obtained the highest viable cell counts (9.89 log CFU/mL) in 24 h of incubation, and it was able to maintain higher viable cell counts for a longer period of incubation. 

As shown in Figure 1, it was found that with ultrasonic treatment at 60 °C for 3 h on the Oka medium before inoculation, the β-glucosidase activity of *Lp*-BCRC10357 at 12 h and 24 h of incubation amounted to 13.35 and 15.50 U/mL, respectively, which was higher and more stable than that (12.58 U/mL at 12 h and 2.86 U/mL at 24 h) without ultrasonic treatment of the medium. This implied that the use of different conditions of ultrasonic treatment (40 kHz/300 W) on the Oka medium could cause changes in the structure of the okara, which could lead to differences in the growth behaviors of *Lp*-BCRC10357 due to the utilization of nutrients from the okara. Yu and Yang (2019) reported that ultrasonic treatment helps to enhance the openness of the microstructure of the okara [16].

Su et al. (2023) indicated that the survivability of LAB can be enhanced by the limited strength of nutrients and ultrasonic stresses [18]. Aiello et al. (2021) reported that ultrasound could be applied to effectively extract residual proteins with conformational changes from okara by-products [15], and such extracted proteins might be utilized by bacteria as nutrients during fermentation. In this study, the ultrasonic treatment at 40 kHz/300 W allowed us to modify the okara structure to facilitate the transport of ingredients and bacteria. A more severe condition of ultrasonic treatment at 60 °C for 3 h on the medium (Oka-US) was more favorable for the survival and β-glucosidase activity of *Lp*-BCRC10357.

### 3.2. Effect of the Amount of ChCl Added to Black Soybean Okara Medium

For the Oka medium with various amounts of ChCl (1, 3, or 5%) under ultrasonic treatment at 60 °C for 3 h, the viable cell counts of *Lp*-BCRC10357 during 48 h of incubation at 37 °C are shown in Table 2. It was found that during 48 h of incubation, the viable cell counts for 1%ChCl-US remained at a higher level compared with those for other cases. In 12 h of incubation, the viable cell count for 1%ChCl-US quickly rose to 9.81 log CFU/mL, and it was significantly higher than those for 3%ChCl-US (9.54 log CFU/mL) and 5%ChCl-US (9.57 log CFU/mL). Different amounts of ChCl present in the growth medium led to different extracellular environments, resulting in various degrees of stress on *Lp*-BCRC10357. Such stress induced *Lp*-BCRC10357 to regulate its physiological status to adapt to the new environment. The results showed that a limited quantity of ChCl added to the Oka medium could elicit a significant positive response of *Lp*-BCRC10357. 

**Figure 2:**
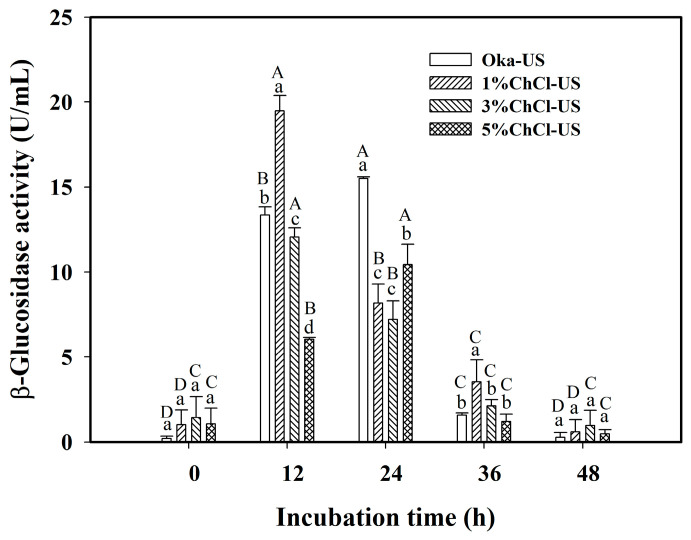
Effect of different amounts of choline chloride (ChCl) added to the medium of MRS with 3% black soybean okara (Oka) on the β-glucosidase activity of *L. plantarum* BCRC10357 incubated at 37 °C for 48 h. Oka-US: the Oka medium with ultrasonic treatment (40 kHz/300 W) at 60 °C for 3 h before inoculation; x%ChCl-US: the Oka medium combined with x% ChCl using ultrasonic treatment (40 kHz/300 W) at 60 °C for 3 h before inoculation. Data were expressed as mean ± standard deviation (*n* = 3). Different superscript lowercase letters at the same incubation time and different superscript uppercase letters at the same amounts of ChCl were significantly different (*p* < 0.05) by Duncan’s multiple range test.

Figure 2 shows the results of the β-glucosidase activity of *Lp*-BCRC10357 for the Oka medium combined with different amounts of ChCl under ultrasonic treatment (40 kHz/300 W) at 60 °C for 3 h, and the results for Oka-US (without adding ChCl) are also displayed for comparison. It shows that in 12 h of incubation, the β-glucosidase activity for 1%ChCl-US quickly rose to 19.47 U/mL, being significantly larger than those for other cases with the order of 1%ChCl-US > Oka-US > 3%ChCl-US > 5%ChCl-US. This showed that the addition of 1% ChCl to the Oka medium with ultrasonic treatment could stimulate the bacteria to further release β-glucosidase in a short incubation time, compared with the case of Oka-US (no ChCl), for which the largest β-glucosidase activity (15.50 U/mL) occurred at 24 h of incubation. 

Furthermore, for the case of 5%ChCl-US, the largest β-glucosidase activity (10.44 U/mL) of *Lp*-BCRC10357 occurred at 24 h of incubation, revealing that the reduction in β-glucosidase activity could be delayed by adding a suitable amount of ChCl to the medium. However, the results shown in Figure 2 demonstrate that a large amount of ChCl added to the medium was not essential for the positive response of *Lp*-BCRC10357. The reason might be related to the role of the ChCl structure in the solution environment. Nanavare et al. (2022) reported that an increase in the amount of ChCl could lead to a disruption in the tetrahedrality for water molecules, with a reduction in hydrogen bonds between water pairs in the solution [28]. Maity et al. (2020) concluded that the bulky choline ion can be considered to act as a nano-crowder that could suppress the dynamics of the proteins as well as other co-solvents to prevent the unfolding of proteins [29]. Therefore, a limited amount of ChCl in the medium was sufficient to induce a favorable response regarding the release of β-glucosidase for fermentation. Furthermore, the β-glucosidase activity of *Lp*-BCRC10357 for 1%ChCl-US at 12 and 24 h was much greater than that for the Oka (no US), showing that the combination of 1% ChCl with ultrasonic treatment could obtain the best positive response of *Lp*-BCRC10357 in the fermentation of black soybean okara.

### 3.3. Effect of Different Modes of Ultrasonic Treatment for the Medium System Using 1% ChCl

**Figure 3:**
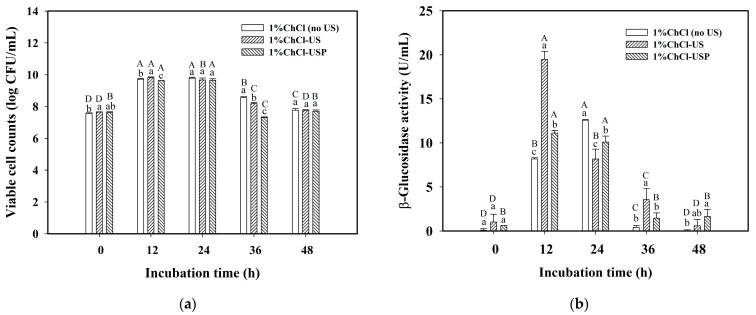
Effect of various modes of ultrasonic treatment on (**a**) viable cell counts; (**b**) β-glucosidase activity of *L. plantarum* BCRC 10357 incubated at 37 °C for 48 h. Oka: the medium of MRS with 3% black soybean okara; 1%ChCl (no US): the Oka medium combined with 1% choline chloride (ChCl) without ultrasonic treatment; 1%ChCl-US: the Oka medium with ultrasonic treatment (40 kHz/300 W) at 60 °C for 3 h before inoculation. 1%ChCl-USP: the medium of 1%ChCl-US using *L. plantarum* BCRC 10357 that had been pretreated with an ultrasound probe (20 kHz/200 W) at 60% amplitude and 25 °C for 2 min. Data were expressed as mean ± standard deviation with triplicate experiments (*n* = 3). Different superscript lowercase letters at the same incubation time and different superscript uppercase letters at the same modes of ultrasonic treatment were significantly different (*p* < 0.05) by Duncan’s multiple range test.

The effect of different modes of ultrasonic treatment for the Oka medium containing 1% ChCl on the survivability of *Lp*-BCRC10357 was explored, and three cases were compared, i.e., (1) the medium without ultrasonic treatment (1%ChCl (no US)), (2) the medium with ultrasonic treatment (1%ChCl-US), and (3) the medium with ultrasonic treatment and using the *Lp*-BCRC10357 pretreated with an ultrasound probe before its inoculation (1%ChCl-USP). The results for the viable cell counts and β-glucosidase activity are shown in Figure 3a,b, respectively. It was found that, in a short incubation time of 12 h, the viable cell counts for 1%ChCl-US (9.81 log CFU/mL) were significantly higher than those for 1%ChCl (no US) (9.71 log CFU/mL) and 1%ChCl-USP (9.62 log CFU/mL) (Figure 3a), and the order of β-glucosidase activity was 1%ChCl-US (19.47 U/mL) > 1%ChCl-USP (11.11 U/mL) > 1%ChCl (no US) (8.18 U/mL). 

Although the β-glucosidase activity for 1%ChCl (no US) could increase to a value of 12.57 U/mL in 24 h of incubation, it quickly diminished to 0.42 U/mL in 36 h of incubation. In contrast, with 1% ChCl in the Oka medium using ultrasonic treatment, either with or without bacteria to be pretreated, the β-glucosidase activity quickly rose to a higher value in 12 h of incubation, delaying any reduction, compared to that for 1%ChCl (no US). This demonstrates that the use of 1%ChCl-US could provide a more beneficial environment for the growth and physiological adaptation of *Lp*-BCRC10357 than the use of 1%ChCl-USP.

### 3.4. Antioxidant Activity of the Fermented Product

**Figure 4:**
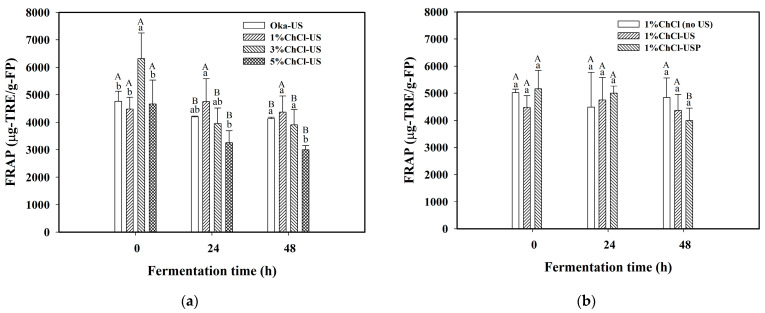
Variations in ferric-reducing antioxidant power (FRAP) in the dried fermented product (FP) at 0, 24, and 48 h of fermentation time for the effects of (**a**) amount of choline chloride (ChCl) added to the Oka medium and (**b**) mode of ultrasonic treatment on the medium or on *L. plantarum* BCRC 10357. Oka: the medium of MRS with 3% black soybean okara; Oka-US: the Oka medium with ultrasonic treatment (40 kHz/300 W) at 60 °C for 3 h before inoculation; 1%ChCl (no US): the Oka medium combined with 1% ChCl without ultrasonic treatment; x%ChCl-US: the Oka medium combined with x% ChCl using ultrasonic treatment (40 kHz/300 W) at 60 °C for 3 h before inoculation; 1%ChCl-USP: the medium of 1%ChCl-US inoculated with *L. plantarum* BCRC 10357 pretreated with ultrasound probe (20 kHz/200 W) at 25 °C for 2 min. Data were expressed as mean ± standard deviation (*n* = 3). Different superscript lowercase letters at the same fermentation time and different superscript uppercase letters at the same amounts of ChCl for (**a**) or at the same modes of ultrasonic treatment for (**b**) were significantly different (*p* < 0.05) by Duncan’s multiple range test.

The effects of ultrasonic treatment and ChCl addition on the antioxidant activity of the fermented product in the fermentation of black soybean okara using *Lp*-BCRC10357 were evaluated. Figure 4a displays the comparison of the ferric-reducing antioxidant power (FRAP) of the dried fermented product for different amounts of ChCl in the Oka medium with ultrasonic treatment. In 24 h of fermentation, the FRAP for 1%ChCl-US was significantly higher than that for other cases in the order of 1%ChCl-US (4749.03 μg-TRE/g-FP) > Oka-US (4195.68 μg-TRE/g-FP) > 3%ChCl-US (3951.05 μg-TRE/g-FP) > 5%ChCl-US (3256.13 μg-TRE/g-FP). For the case of 1%ChCl-US, the FRAP in the fermented product increased from 4479.11 μg-TRE/g-FP at 0 h to 4749.03 μg-TRE/g-FP at 24 h, and then slightly decreased to 4368.27 μg-TRE/g-FP at 48 h of fermentation. Meanwhile, for both the 3%ChCl-US and 5%ChCl-US cases, the FRAP values first decreased in 24 h of fermentation and further slightly decreased with 48 h of fermentation. 

This trend of a decrease in FRAP during fermentation was also observed for Oka-US but with a smaller rate of decrease. The changes in antioxidant activity might have resulted from the changes in the content of functional ingredients after fermentation, such as the content of isoflavones and phenolic compounds, because the responses of *Lp*-BCRC10357 might vary when experiencing different environmental stresses. Chen et al. (2022) reported that the antioxidant activity was substantially related to the contribution of the phenolic compounds [30]. Gupta et al. (2018) indicated that the increase in antioxidant activity in okara after fermentation might be attributed to an increase in the content of phenolics [31]. Wang et al. (2022) reported that the antioxidant activity of the okara samples decreased after 24 h of fermentation using *L. plantarum* UFG169, and this was related to the decrease in isoflavone and B_2_ content [12]. In this study, the limited presence of 1% ChCl in the Oka medium was sufficient to enhance the antioxidant activity of the fermented product in 24 h of fermentation.

Figure 4b displays the comparison of the antioxidant activity (in FRAP) between different ultrasonic modes using 1% ChCl in the Oka medium for the cases of 1%ChCl (no US), 1%ChCl-US, and 1%ChCl-USP. As shown in Figure 4b, in 24 h of fermentation, the order of FRAP was 1%ChCl-USP > 1%ChCl-US > 1%ChCl (no US), but with an insignificant difference; meanwhile, in 48 h of fermentation, the order of FRAP was reversed as 1%ChCl (no US) > 1%ChCl-US > 1%ChCl-USP, still with an insignificant difference. However, the case of 1%ChCl-USP exhibited a larger reduction in antioxidant activity after fermentation, and a more stable profile of antioxidant activity was obtained for 1%ChCl-US.

### 3.5. Biotransformation of Isoflavones in the Fermentation of Black Soybean Okara Containing Choline Chloride

The cases without/with ultrasonic treatment (40 kHz/300 W) at 60 °C for 3 h and the effects of different amounts of ChCl (1%, 3%, and 5%) in the Oka medium on the biotransformation of isoflavones in the fermentation of black soybean okara using *Lp*-BCRC10357 were evaluated. The results are shown in Table 3. 

The enhancement ratio was used to assess the efficiency of the biotransformation of isoflavone glycosides to their aglycones, and it was defined as (AI/TI) at a fermentation time of 24 h or 48 h divided by (AI/TI) at a fermentation time of 0 h, where AI was the sum of daidzein and genistein and TI was the sum of daidzin, genistin, daidzein, and genistein. The enhancement ratio decreased with the increase in %ChCl in the Oka medium under ultrasonic treatment, and the order of the enhancement ratio was 1%ChCl-US (5.54) > 3%ChCl-US (4.03) > 5%ChCl-US (2.33) at 24 h of fermentation. The same trend was observed at 48 h of fermentation. This demonstrated that the excessive addition of ChCl would lead to changes in the medium environment, not only inducing different responses of *Lp*-BCRC10357 in releasing β-glucosidase but also affecting the biotransformation of the ingredients by the enzymes, due to ChCl acting as an osmoprotectant and nano-crowder that could suppress the dynamics of the protein [29]. When using 1% ChCl in the Oka medium, the enhancement ratios with ultrasonic treatment (1%ChCl-US) were much higher than those without ultrasonic treatment (1%ChCl (no US)) at both 24 h and 48 h of fermentation, indicating that the modification of the surface structure of solid black soybean okara using ultrasound could facilitate the transport of the components and bacteria into the network structure for fermentation.

### 3.6. Morphological Structures of the Solid Substrate with L. plantarum BCRC 10357

**Figure 5:**
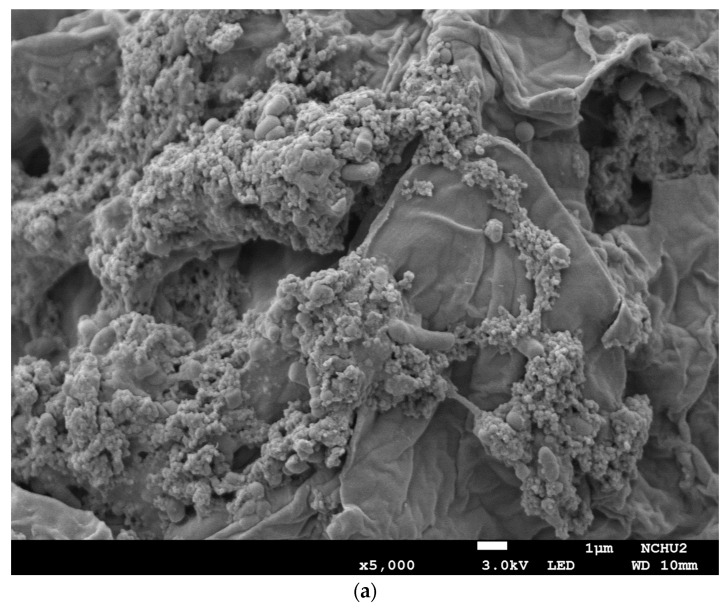

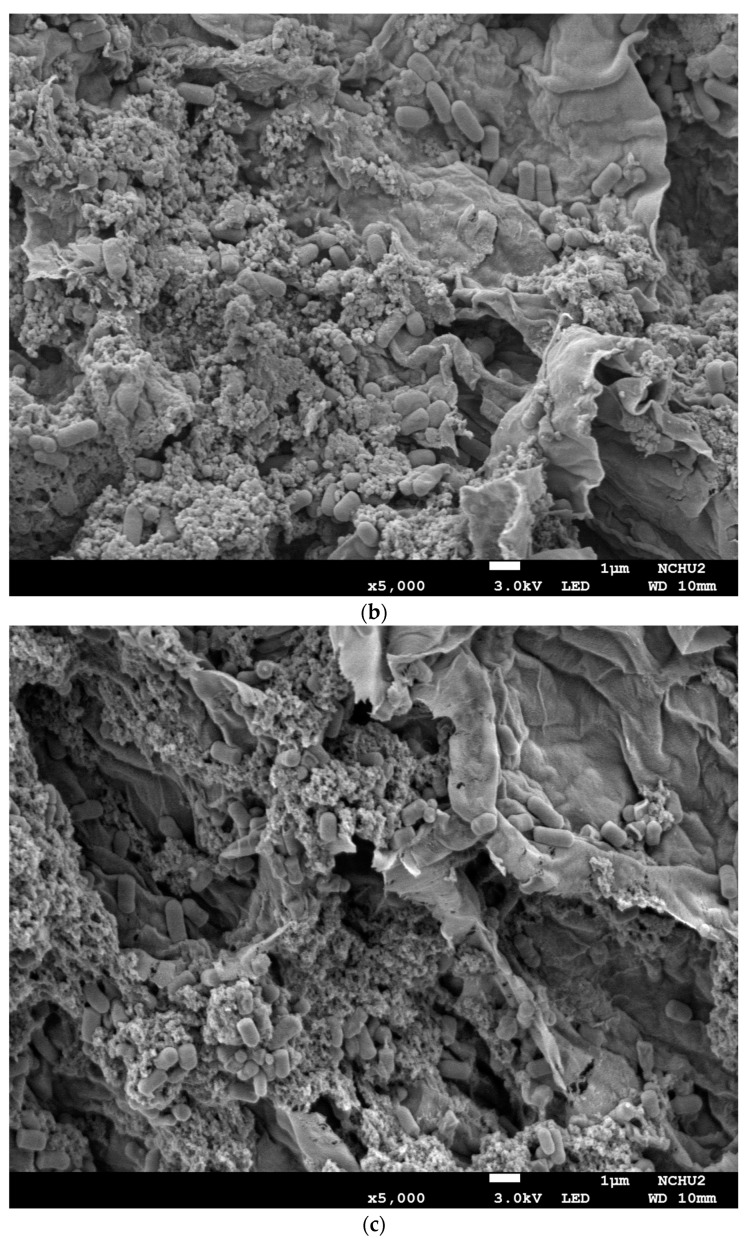

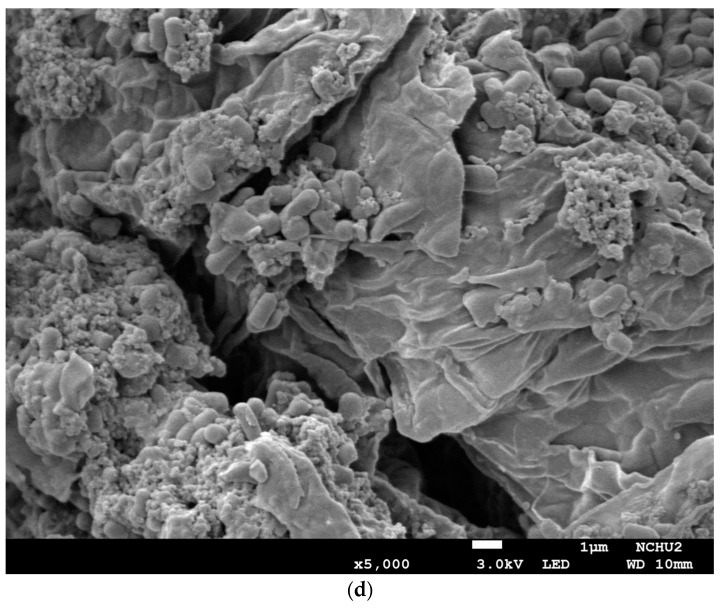
FE-SEM images of the solids separated from the medium in 12 h with *L. plantarum* BCRC 10357: (**a**) Oka-US; (**b**) 1%ChCl (no US); (**c**) 1%ChCl-US; (**d**) 1%ChCl-USP. Oka: the medium of MRS combined with 3% black soybean okara; Oka-US: the Oka medium with ultrasonic treatment (40 kHz/300 W) at 60 °C for 3 h before inoculation; 1%ChCl (no US): the Oka medium combined with 1% choline chloride (ChCl) without ultrasonic treatment; 1%ChCl-US: the Oka medium combined with 1% ChCl using ultrasonic treatment (40 kHz/300 W) at 60 °C for 3 h before inoculation; 1%ChCl-USP: the medium of 1%ChCl-US using *L. plantarum* BCRC 10357 that had been pretreated with an ultrasound probe (20 kHz/200 W) at 25 °C for 2 min.

Figure 5 shows the FE-SEM images of the solid substrate separated from the medium in 12 h with *Lp*-BCRC10357. As shown in Figure 5a, for Oka-US, after ultrasonic treatment, the structure of the okara was destroyed to some extent, leading to an enhancement in the transport of ingredients and bacteria, approaching the internal surface more easily.

Figure 5b displays the surface morphology for 1%ChCl (no US). Without ultrasonic treatment but with 1% ChCl in the Oka medium, the surface of the okara was more flat and less broken, showing that the bacteria were very crowded, gathering on the surface of the okara. In Figure 5c, for the microstructure of 1%ChCl-US, with ultrasonic treatment of the okara and 1% ChCl, the surface of the okara was much destroyed and broken to expose the internal sites, and the bacteria were able to easily enter the internal structure for fermentation. In Figure 5d, for 1%ChCl-USP, it is shown that the structure of the okara was broken but still had some flat surfaces, and the pretreated bacteria were clearly gathered on the surface.

## 4. Conclusions

In this study, the impacts of ultrasonic treatment for the culture medium containing solid black soybean okara and ChCl on the survival and β-glucosidase activity of *Lp*-BCRC10357 were assessed. The presence of 1% ChCl in the Oka medium with ultrasonic treatment could stimulate the best response of *Lp*-BCRC10357 to release the most β-glucosidase at 12 h of incubation. The intense ultrasonic treatment caused the surface structure of the solid black soybean okara to be severely destroyed, so as to facilitate the transport of ingredients and *Lp*-BCRC10357 into the internal structure of the okara for the fermentation. Moreover, the positive response of *Lp*-BCRC10357 was induced by regulating its physiological behavior when confronting the extra added ChCl, which acted as an osmoprotectant and nano-crowder in the extracellular environment. This study developed a methodology by combining ultrasonic treatment with a limited amount of ChCl in the culture medium of black soybean okara to acclimate *Lp*-BCRC10357 to release a high level of β-glucosidase, and this approach has the potential to be used in the fermentation of okara-related products as a nutritional supplement in foods, for the valorization of okara.

## Figures and Tables

**Table 1:** Effect of different conditions of ultrasonic treatment (40 kHz/300 W) for the medium of MRS combined with 3% black soybean okara (Oka) on viable cell counts of *L. plantarum* BCRC 10357.

Ultrasonic Treatment	Viable Cell Count (log CFU/mL) at Incubation Time (h) *				
	0	12	24	36	48
Oka (no US)	7.62 ± 0.04 ^aC^	9.59 ± 0.01 ^bA^	9.41 ± 0.01 ^cB^	6.99 ± 0.02 ^bD^	6.60 ± 0.06 ^bE^
Oka-US-L	7.61 ± 0.02 ^aB^	9.58 ± 0.01 ^bA^	9.68 ± 0.03 ^bA^	7.07 ± 0.08 ^abC^	6.11 ± 0.12 ^cD^
Oka-US	7.53 ± 0.04 ^bD^	9.74 ± 0.04 ^aB^	9.89 ± 0.03 ^aA^	7.17 ± 0.03 ^aE^	8.01 ± 0.09 ^aC^

* Conditions: incubation at 37 °C for 48 h. Oka (no US): the Oka medium without ultrasonic treatment; Oka-US-L: the Oka medium with ultrasonic treatment at 30 °C for 20 min before inoculation; Oka-US: the Oka medium with ultrasonic treatment at 60 °C for 3 h before inoculation. Data were expressed as mean ± standard deviation with triplicate experiments (*n* = 3). Different superscript lowercase letters in the same columns and different superscript uppercase letters in the same rows were significantly different (*p* < 0.05) by Duncan’s multiple range test.

**Table 2:** Effect of different amounts of choline chloride (ChCl) added to the medium of MRS with 3% black soybean okara (Oka) under ultrasonic treatment (40 kHz/300 W) on viable cell counts of *L. plantarum* BCRC 10357.

Type of Medium	Viable Cell Count (log CFU/mL) at Incubation Time (h) *				
	0	12	24	36	48
1%ChCl-US	7.65 ± 0.02 ^abD^	9.81 ± 0.05 ^aA^	9.66 ± 0.13 ^aB^	8.18 ± 0.06 ^aC^	7.76 ± 0.03 ^aD^
3%ChCl-US	7.71 ± 0.04 ^aB^	9.54 ± 0.03 ^bA^	9.56 ± 0.12 ^aA^	7.46 ± 0.05 ^bC^	6.86 ± 0.09 ^bD^
5%ChCl-US	7.60 ± 0.05 ^bC^	9.57 ± 0.09 ^bA^	9.69 ± 0.02 ^aA^	8.18 ± 0.08 ^aB^	6.54 ± 0.09 ^cD^

* Conditions: incubation at 37 °C for 48 h. x%ChCl-US: the Oka medium combined with x% ChCl using ultrasonic treatment at 60 °C for 3 h before inoculation. Data were expressed as mean ± standard deviation with triplicate experiments (*n* = 3). Different superscript lowercase letters in the same columns and different superscript uppercase letters in the same rows were significantly different (*p* < 0.05) by Duncan’s multiple range test.

**Table 3:** Effects of ultrasonic treatment in the presence of various amounts of choline chloride (ChCl) on the biotransformation of isoflavones in the dried fermented product (FP) for the fermentation of black soybean okara using *L. plantarum* BCRC 10357 at 37 °C *.

Medium Type	Fermentation Time (h)	Isoflavones (μg/g-FP)				AI/TI (%)	Enhancement Ratio
		Daidzin	Genistin	Daidzein	Genistein		
1%ChCl (no US)	0	86.73 ± 2.53 ^A^	104.98 ± 6.21 ^A^	15.71 ± 0.97 ^B^	18.34 ± 1.33 ^B^	15.08	--
1%ChCl (no US)	24	43.12 ± 31.00 ^B^	59.73 ± 28.77 ^B^	28.47 ± 9.82 ^A^	29.77 ± 6.88 ^A^	36.15	2.40
1%ChCl (no US)	48	27.75 ± 2.05 ^B^	54.50 ± 0.86 ^B^	33.16 ± 1.43 ^A^	29.45 ± 0.88 ^A^	43.22	2.87
1%ChCl-US	0	108.12 ± 4.48 ^A^	152.23 ± 2.31 ^A^	9.51 ± 0.62 ^B^	9.75 ± 1.44 ^C^	6.89	--
1%ChCl-US	24	31.22 ± 2.89 ^B^	65.57 ± 1.25 ^B^	33.45 ± 1.26 ^A^	26.25 ± 1.34 ^A^	38.15	5.54
1%ChCl-US	48	28.01 ± 3.54 ^B^	74.15 ± 20.84 ^B^	30.48 ± 2.73 ^A^	21.97 ± 2.32 ^B^	33.92	4.92
3%ChCl-US	0	89.21 ± 10.30 ^A^	132.59 ± 18.44 ^A^	9.56 ± 1.49 ^B^	10.08 ± 1.22 ^C^	8.13	--
3%ChCl-US	24	27.13 ± 0.22 ^B^	66.95 ± 12.90 ^B^	25.47 ± 1.66 ^A^	20.32 ± 1.86 ^A^	32.74	4.03
3%ChCl-US	48	28.06 ± 0.51 ^B^	54.46 ± 2.91 ^B^	23.97 ± 1.40 ^A^	17.15 ± 1.57 ^B^	33.26	4.09
5%ChCl-US	0	58.51 ± 0.48 ^A^	69.72 ± 1.72 ^A^	10.09 ± 0.36 ^C^	11.92 ± 0.13 ^C^	14.65	--
5%ChCl-US	24	29.82 ± 0.19 ^B^	41.70 ± 0.35 ^B^	18.60 ± 0.10 ^B^	18.38 ± 0.79 ^B^	34.09	2.33
5%ChCl-US	48	21.89 ± 2.03 ^C^	30.30 ± 0.75 ^C^	21.51 ± 0.13 ^A^	20.11 ± 0.78 ^A^	44.36	3.03

* Data were expressed as mean ± standard deviation (*n* = 3). AI = daidzein + genistein, TI = daidzin + genistin + daidzein + genistein. (AI/TI)(%) = (AI/TI) × 100%. Enhancement ratio = (AI/TI at time 24 h or 48 h)/(AI/TI at time 0 h). Oka: the medium of MRS with 3% black soybean okara; 1%ChCl (no US): the Oka medium combined with 1% ChCl without ultrasonic treatment; x%ChCl-US: the Oka medium combined with x% ChCl using ultrasonic treatment (40 kHz/300 W) at 60 °C for 3 h before inoculation. Different superscript uppercase letters in the same columns in the same medium type were significantly different (*p* < 0.05) by Duncan’s multiple range test.

## Data Availability

Data is contained within the article.
